# Nonconvulsive status epilepticus: An unusual cause of postoperative unresponsiveness following general anaesthesia

**DOI:** 10.4103/0019-5049.79901

**Published:** 2011

**Authors:** P Sudha, Rachel Cherian Koshy

**Affiliations:** Division of Anaesthesiology, Regional Cancer Centre Thiruvananthapuram, Kerala, India

**Keywords:** Nonconvulsive status epilepticus, postoperative unresponsiveness, anaesthetic emergencies

## Abstract

Any altered behaviour or sensorium following general anaesthesia is of concern to the anaesthesiologist, as it could be attributed to the anaesthetic itself or to a hypoxic insult, both of which can have medicolegal implications. It is important to be aware of a relatively unfamiliar entity known as nonconvulsive status epilepticus in this context. We report two cases to highlight this condition.

## INTRODUCTION

Unresponsiveness after emergence from general anaesthesia is a medical emergency. Nonconvulsive status epilepticus (NCSE) as a postoperative phenomenon is a rare cause for unresponsiveness in the postoperative period. This condition is usually not thought of by anaesthesiologists and needs high level of suspicion. It is diagnosed by exclusion and has good prognosis.[1,2]

## CASE REPORTS

### Case 1

A 58-year-old laryngectomised man, weighing 60 kg, was posted for tracheo-oesophageal voice prosthesis implantation. He had type 2 diabetes mellitus well controlled with oral hypoglycaemic agents. He was a non-smoker, a non-alcoholic, with no psychiatric illness or drug addiction. Preoperative vitals and biochemical parameters were within normal limits. He was premedicated with oral alprazolam 0.5 mg and pantoprazole 40 mg the night before and on the day of surgery. Additional premedication was given with intravenous fentanyl 100 mcg and glycopyrrolate 0.2 mg. General anaesthesia was induced with propofol 100 mg. A flexometallic tube of internal diameter 8 mm was introduced through the tracheostomy. Anaesthesia was maintained with air, oxygen and isoflurane; and muscle relaxation, with vecuronium 5 mg. Intraoperative haemodynamic parameters were maintained within normal limits, and hypothermia was prevented using a body warmer. Surgery lasted for 45 minutes. One litre of normal saline was infused intraoperatively.

On complete recovery from anaesthesia, 60 minutes after induction, trachea was extubated after reversing the residual neuromuscular block with neostigmine 2.5 mg and glycopyrrollate 0.4 mg. After extubation, the patient was well oriented, awake and responded to verbal commands. About 10 minutes after extubation, he had a bout of cough. Following this, he was found unresponsive even to intense painful stimuli. He stopped spontaneous respiratory efforts and was re-intubated and ventilated with 100% oxygen. Blood pressure was 200/120 mm Hg and was managed with slow intravenous labetolol 20 mg. Bladder was catheterized and 450 ml of clear urine was drained, Pupils were bilaterally constricted but reacting to light. Body temperature was normal. Hypoglycaemia and electrolyte imbalances were ruled out (RandomBloodSugar,163mg/dl;Serum sodium/pottassium/calcium/magnesium/chloride, 138/4.2/9/2.1/110 mEq/L). Arterial blood gas showed mild hypoxia (pO_2_, 88 mm Hg). Residual neuromuscular blockade was ruled out using a nerve stimulator.

After about 10 minutes, the patient regained spontaneous respiratory attempts; but all other motor and higher cortical functions were absent. There was no facial asymmetry or signs of meningeal irritation. Over the next 20 minutes, he started responding to call with gradual return of motor activity to full-grade power. He appeared slightly disoriented. Neurology consultation was done. Cranial computerized tomography (CT) scan was normal. Electro encephalogram (EEG) was unavailable in our institution and not done. The diagnosis was NCSE, after excluding other causes. The patient was treated successfully with intravenous lorazapam 2 mg and phenytoin 1000 mg.

### Case 2

A 71-year-old man, weighing 65 kg, with carcinoma stomach underwent partial gastrectomy under combined general anaesthesia and lumbar epidural block. He was hypertensive and diabetic, both conditionswell controlled with Angiotensin Converting Enzyme inhibitors and insulin. He had cervical laminectomy and enucleation of schwannoma at C7 level 2 years ago, following which he developed total blindness of left eye due to central retinal artery occlusion. There s weakness of left triceps (power, 3/5) and wasting of left hand muscles. Magnetic resonance imaging and magnetic resonance angiography brain were normal; and vertebral Doppler, negative. He had no mental illness or addiction.

Epidural catheter was inserted at T12-L1 level, and 15 ml of 0.25% bupivacaine with fentanyl 100 mcg was given. General anaesthesia was induced with propofol 100 mg. Vecuronium 6 mg was used for endotracheal intubation and muscle relaxation. Anaesthesia was maintained with air, oxygen and isoflurane. Hypothermia was prevented using body warmer. One thousand two hundred fifty millilitres of normal saline was infused intraoperatively. Hourly urine output was 30-50 ml. Surgery lasted 120 minutes. Trachea was extubated 130 minutes after induction, after reversing the residual neuromuscular blockade with neostigmine 2.5 mg and glycopyrrolate 0.4 mg. Vitals and haemodynamic parameters were within normal limits.

Nearly 24 hours after surgery, the patient developed alteration in sensorium. He remained unresponsive, with a ‘staring’ look but with no seizures. There was no response even to painful stimuli. The unresponsiveness lasted less than 30 minutes, and the patient returned to normal mentation. There was no facial asymmetry. Haemodynamic parameters were within normal limits, but peripheral oxygen saturation was low. There were clinical and radiological signs of left lobar consolidation. Respiration was shallow and was supported with oxygen 5 litres/minute. Laryngeal mask airway was used as there was no proper mask fit as the patient was edentulous. He moved all the limbs symmetrically. There were no meningeal signs. Plantar reflex was normal. Hypoglycaemia and electrolyte abnormalities were ruled out (RBS, 158mg/dl; Na/K/Cl/Mg, 140/3.8/107/2.2mEq/L). Arterial blood gas revealed hypoxia with respiratory acidosis (pO2/pCO2/pH/HCO3, 51 mm Hg/53.6 mm Hg/7.31/26.1 mEq/l; and BE, 1.4 mmol/l). Neuroimaging ruled out cerebrovascular accident. EEG was unavailable in our institution and not done. A diagnosis of NCSE was made by the neurologist after excluding other causes. His neurological abnormality resolved completely with intravenous phenytoin 1000 mg and lorazapam 2 mg, and pneumonia was treated successfully with antibiotics.

## DISCUSSION

If a previously responsive patient develops acute onset of unconsciousness after emergence from anaesthesia, it is very urgent to determine an immediate differential diagnosis. Many studies emphasise the need to keep nonconvulsive status epilepticus in the differential diagnosis of any patient with altered mental status, when the aetiology is unknown. It is an epileptic condition with mental-status changes from baseline with continuous or recurrent seizure activity on the electroencephalogram. The lack of a predominant motor component differentiates this from convulsive status epilepticus.

NCSE may occur in patients with diverse clinical diagnoses, such as hypoxic-anoxic encephalopathy, cancer, autoimmune disorders, drug toxicity, pregnancy, infections, alcohol intoxication/ withdrawal, central nervous system lesions, electroconvulsive therapy, chromosomal alterations, peritoneal dialysis, cerebral hamartomas or head trauma.[3,5] Confirmation of NCSE by EEG is recommended before instituting pharmacological treatment, whenever possible. Spontaneous recovery or immediate response to IV benzodiazepines and conventional antiepileptic drugs (AEDs) is seen in many cases.[6] Once the diagnosis of NCSE is determined, long-term treatment with AED should be considered,[7] as episodes of NCSE often recur.

Patient 1 had met the conditions for extubation — fully awake, responding, adequate muscle power and respiration. He was maintaining 100% saturation until he became completely comatosed with apnoea. The foreign body — voice prosthesis in the airway — would have provoked the spasmodic cough causing some hypoxia, as shown by subsequent arterial blood gas analysis. Among the causes of NCSE, this patient had two positive aetiological factors — cancer and transient relative hypoxia. Good response to anticonvulsants is also in favour of diagnosis of NCSE. If it was relative opioid toxicity/overdose, he would not have become fully awake initially with normal respiration. A dose of 1.6 mcg of fentanyl is well within the normal recommended dose. A trial of naloxone 400 mcg, slowly, intravenously did not produce the immediate dramatic response that is typical of opioid overdose.

The onset of the symptoms in patient 2 is much later (after 24 hours). The patient manifested bizarre neurological symptoms — aphasia with unresponsiveness and a ‘staring’ look. This patient did not have respiratory arrest. The spectrum of clinical manifestations in NCSE ranges from minimal confusion to bizarre behavioural manifestations to psychosis and coma.[8] The onset can be sudden and gradual, and duration can vary from minutes to days or months.[9] Patient’s chest x-ray showed left lower lobe consolidation. He had 2 positive aetiological factors said to be associated with NCSE – cancer and relative hypoxemia due to consolidation. Prompt response to anticonvulsants and lack of motor component favour a diagnosis of NCSE.

## CONCLUSION

Loss of consciousness following general anaesthesia in a previously responsive patient most likely reflects a life-threatening condition that is acutely affecting the patient’s CNS function. NCSE is a possible but often unrecognized cause. Early diagnosis of NCSE needs a high level of suspicion. The diagnosis and treatment of NCSE should be performed early due to the potential brain damage associated with it.

## References

[1] Kaplan PW (1999). Assessing the outcomes in patients with nonconvulsivestatus epilepticus: Nonconvulsive status epilepticus is under diagnosed, potentially over treated, and confounded by comorbidity. J Clin Neurophysiol.

[2] Murthy JM (2003). Nonconvulsive status epilepticus: An under diagnosed and potentially treatable condition. Neurol India.

[3] Lugaresi E, Pazzaglia P, Tassinari C (1971). Differentiation of absence status and temporal lobe status. Epilepsia.

[4] Thomas P, Beaumanoir A, Genton P, Dolisi C, Chatel M (1992). ‘De novo’ absence status of late onset: Report of 11 cases. Neurology.

[5] Povlsen UJ, Wildschiødtz G, Høgenhaven H, Bolwig TG (2003). Nonconvulsive status epilepticus after electroconvulsive therapy. J ECT.

[6] DeLorenzo RJ, Waterhouse EJ, Towne AR, Boggs JG, Ko D, DeLorenzo GA (1998). Persistent nonconvulsive status epilepticus after control of convulsive status epilepticus. Epilepsia.

[7] Tomson T, Lindbom U, Nilsson B (1992). Nonconvulsive status epilepticus in adults: Thirty-two consecutive patients from a general hospital population. Epilepsia.

[8] Walker M, Cross H, Smith S, Young C, Aicardi J, Appleton R (2005). Nonconvulsive status epilepticus: Epilepsy Research Foundation workshop Reports. Epileptic Disord.

[9] Lowenstein DH, Bleck T, Macdonald RL (1999). It’s time to revise the definition of status epilepticus. Epilepsia.

